# Megaprostheses for the revision of infected hip arthroplasties with severe bone loss

**DOI:** 10.1186/s12893-022-01517-y

**Published:** 2022-02-25

**Authors:** Nicola Logoluso, Francesca Alice Pedrini, Ilaria Morelli, Elena De Vecchi, Carlo Luca Romanò, Antonio Virgilio Pellegrini

**Affiliations:** 1 IRCCS Istituto Ortopedico Galeazzi, Centre for Reconstructive Surgery and Osteoarticular Infections (C.R.I.O. Unit), via Riccardo Galeazzi 4, 20161 Milan, Italy; 2 Residency Program in Orthopaedics and Traumatology, University of Milan, via Festa del Perdono 7, 20122 Milan, Italy; 3 ASST Ovest Milanese, Ospedale di Legnano, UOC Ortopedia e Traumatologia, via Papa Giovanni Paolo II, 20025 Legnano, MI Italy; 4 IRCCS Istituto Ortopedico Galeazzi, Laboratory of Clinical Chemistry and Microbiology, via Riccardo Galeazzi 4, 20161 Milan, Italy; 5 Studio Cecca-Romanò, Corso Venezia 2, 20121 Milan, Italy

**Keywords:** Periprosthetic joint infection, Modular megaprosthesis, Non-oncologic megaprosthesis, Limb salvage, Revision total hip arthroplasty, Proximal femoral arthroplasty, Bone and joint infections, Coatings

## Abstract

**Background:**

Periprosthetic hip infections with severe proximal femoral bone loss may require the use of limb salvage techniques, but no agreement exists in literature regarding the most effective treatment. Aim of this study is to analyze the infection eradication rate and implant survival at medium-term follow-up in patients treated with megaprostheses for periprosthetic hip infections with severe bone loss.

**Methods:**

Twenty-one consecutive patients were retrospectively reviewed at a mean 64-month follow-up (24–120). Functional and pain scores, microbiological, radiological and intraoperative findings were registered. Kaplan Meier survival analysis and log rank test were used for infection free survival and implant survival analyses.

**Results:**

The infection eradication rate was 90.5%, with an infection free survival of 95.2% at 2 years (95%CI 70.7–99.3) and 89.6%(95%CI 64.3–97.3) at 5 years. Only two patients required major implant revisions for aseptic implant loosening. The most frequent complication was dislocation (38.1%). The major revision-free survival of implants was 95.2% (95%CI 70.7–99.3) at 2 years and 89.6% (95%CI 64.3–97.3) at 5 years. The overall implant survival was 83.35% (CI95% 50.7–93.94) at 2 and 5 years. Subgroup analyses (cemented versus cementless MPs, coated versus uncoated MPs) revealed no significant differences at log rank test, but its reliability was limited by the small number of patients included.

**Conclusions:**

Proximal femoral arthroplasty is useful to treat periprosthetic hip infections with severe bone loss, providing good functional results with high infection eradication rates and rare major revisions at medium-term follow-up. No conclusions can be drawn on the role of cement and coatings.

## Introduction

Severe bone loss, either on femoral or acetabular side, is an important issue to be addressed during revision hip arthroplasties. It usually develops secondary to multiple revisions, osteolysis following periprosthetic joint infection (PJI) or aseptic loosening, periprosthetic fractures or patients’ comorbidities [1–6]. Revision total hip arthroplasty (RTHA) could be particularly challenging in case of PJIs, because, beyond the bone loss, two further issues should be faced. The first is infection itself, whose eradication is difficult because of biofilm formation, especially in case of “difficult-to-treat” pathogens [7]. The second is the wide spectrum of systemic diseases affecting the patients, who are often also immunocompromised [8, 9]. This leads to a more complex recovery and may potentially affect the overall outcomes in these cohort of patients [1, 9].

One- or two-stage RTHA are usually indicated to treat late chronic PJIs (Tsukuyama type IV) and are effective in restoring good patient functionality and life quality [10–12]. Nevertheless, in case of severe proximal femoral bone loss, few options exist for limb salvage, such as the use of allograft-prosthesis composites, resection arthroplasties, and proximal femoral arthroplasties (PFA). Regardless of these techniques, currently there is no agreement in the literature as to the most effective treatment [1–4].

To date, few studies reported the use of modular megaprostheses (MP) for PJIs treatment [1, 13–19].

The aim of this study was to retrospectively review our cohort of PJI patients treated with PFA due to severe bone loss, analyzing their outcomes in terms of infection eradication, complication rate and implant survival. We hypothesized that PFA could be a good limb salvage option in these patients, providing an acceptable function, long implant survival and high eradication rates.

## Patients and methods

### Patients and study characteristics

**Fig. 1:**
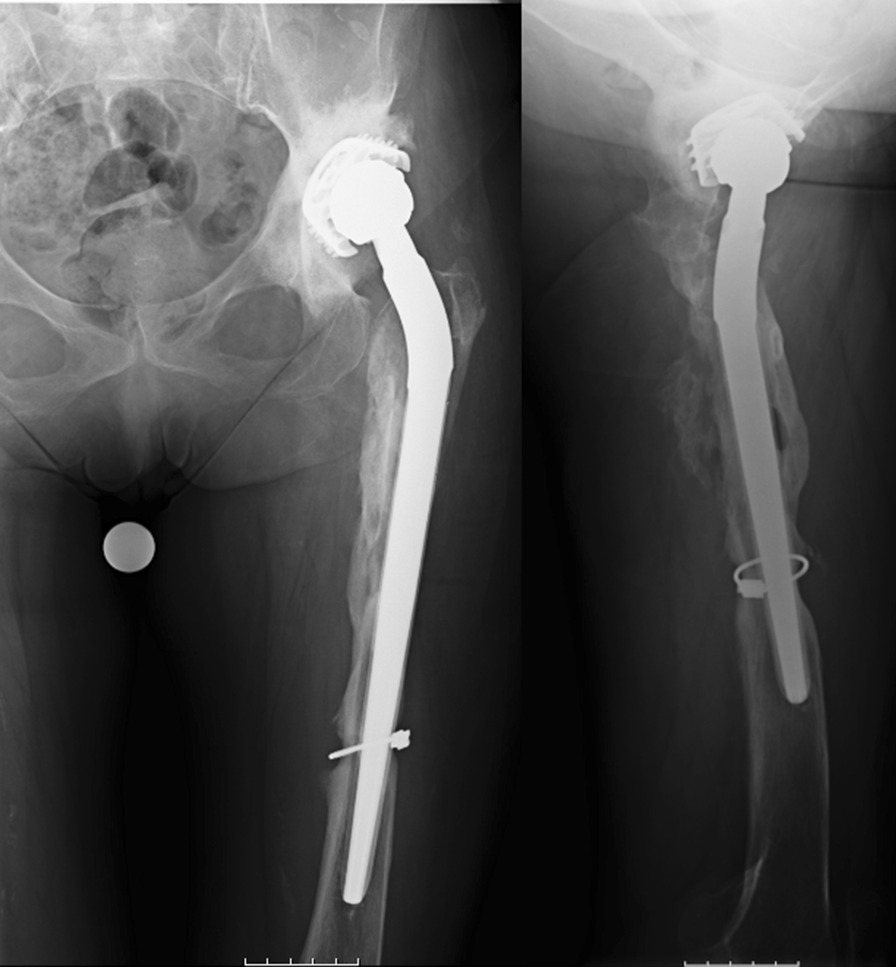
Case no. 20, preoperative X-rays showing the loosened implant surrounded by osteomyelitic bone

In this retrospective cohort study, performed in accordance with STROBE guidelines, we searched our unit database from January 2010 through June 2018, for all consecutive cases of chronic PJIs (Tsukayama type IV) treated with PFA as limb salvage option because the severe femoral bone loss (before or after our radical debridement) contraindicated standard RTHA (Fig. 1) [10].

Diagnosis of infection was made according to the MSIS criteria [20]. We collected data on patient demographics, patient comorbidities, preoperative clinical data, number of previous procedures, the degree of bone loss according to Paprosky’s classifications [21, 22], indications for MP use, preoperative microbiological information whenever available, type of surgery (one- or two-stage procedure), type of MP, type of fixation (cemented or cementless), use of antibacterial coatings, intraoperative microbiological findings, complications, revisions, radiological follow-up according to Harris’ criteria [23]. The search started from 2010, year since when patients’ data became available in the hospital electronic registry.

All patients managed with RTHA of both femoral stem and acetabular component, with a minimum follow-up of 24 months after the final surgery, were included. We excluded patients with any oncologic diagnoses and those who received total femoral replacements.

Then, 21 patients (15 females and 6 males), with a mean age at surgery of 67.9 years (34–90), were included, out of 2816 records screened.

### Surgical technique and post-operative management

All surgeries followed the same surgical protocol. Hardinge’s direct lateral approach was used. The procedure was carried out in one or two stages. According to our internal protocol, one-stage exchange is preferred in “type A” hosts according to McPherson classification, with good tissue coverage, when a single “low-virulence” antibiotic-susceptible causative pathogen was preoperatively known [24, 25]. Two-stage revisions were performed in patients not meeting the above-mentioned criteria, with persistent infections, sinus tracts and antibiotic-resistant microorganisms. Nevertheless, these indications were adapted to the patients’ social and clinical needs.

Paprosky system for acetabular and femoral bone losses was used for decision-making at the time of the reimplantation.

**Fig. 2:**
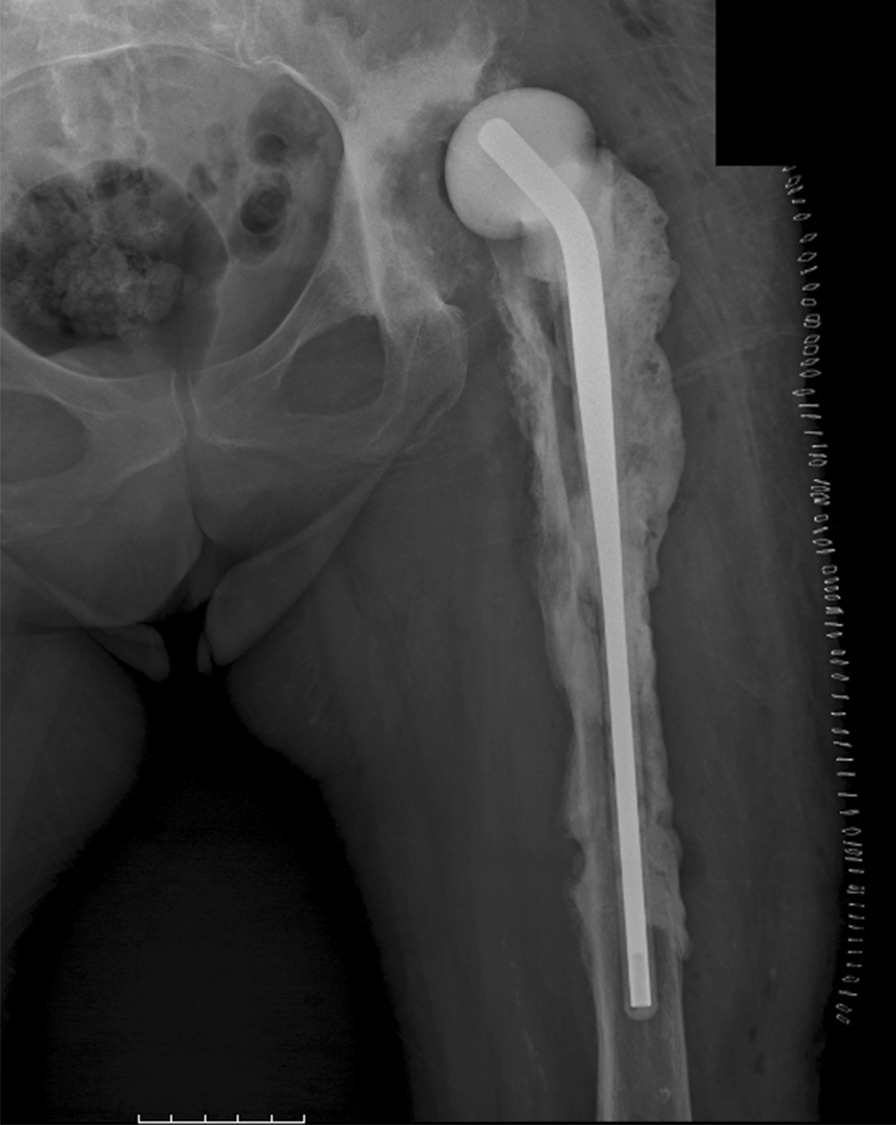
Case no. 20, X-rays showing a subluxated spacer in situ

In two-stage surgeries, the first stage included: implant removal, debridement of any dead tissue, osteomyelitic bone resection. Tissue samples were sent for histology and microbiology (at least 5 specimens, including the removed implant) [26]. After saline irrigation and surgical gloves and drapes change, a preformed long-stem cement spacer, loaded either with gentamicin or vancomycin and gentamicin (respectively, *Spacer-G®* and *Vancogenx®*, Tecres, Sommacampagna, Italy) was introduced (Fig. 2).

The choice between the two spacers was based on the preoperative antibiograms, when available. Alternatively, a molded cement spacer (StageOne™ Select, Biomet Orthopaedics, Warsaw, Indiana, US), which could be loaded intraoperatively with other antibiotics, was preferred when vancomycin- and gentamicin-resistant pathogens had been isolated preoperatively. All patients followed a postoperative antibiotic protocol set by our infectious diseases consultant and based on the intraoperative cultures antibiogram [27].

**Fig. 3:**
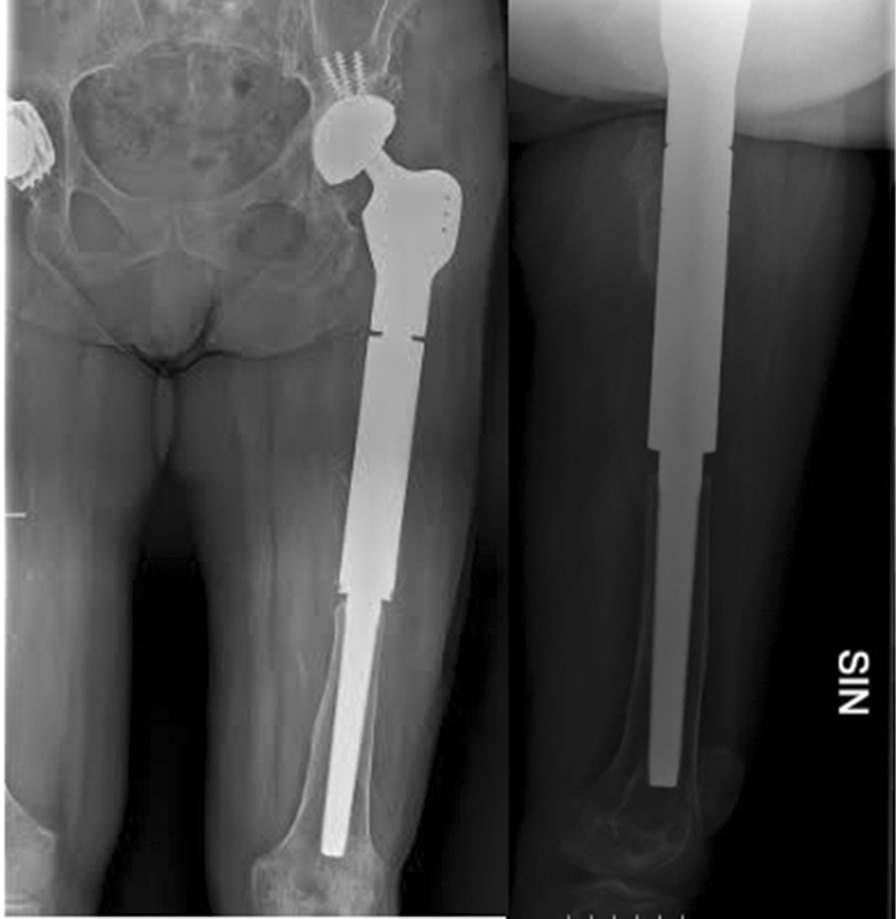
Case no. 20: Postoperative anteroposterior and lateral X-ray of a megaprosthesis

The second stage procedures were performed once PJI was considered eradicated, in patients with no symptoms of infection and a serum C-reactive protein (CRP) < 1 mg/dl after at least fifteen days of antibiotic suspension. During the second stage, the spacer was removed, a new debridement was performed, and at least four tissue samples and the spacer were collected for microbiological investigation. Based on the Paprosky classification, small acetabular bone defects were addressed with cementless standard or multihole revision cups, while in case of massive defects Burch-Schneider cages were chosen. A dual-mobility insert was used in most cases to reduce the dislocation risk. The preferred bearing surfaces were ceramic-on-polyethylene. The femoral mega-implants were either *Mega-C*® *System* (LINK, Hamburg, Germany), cemented or uncemented, according to stem primary stability, or Distally-Interlocked Modular Femoral Reconstruction Prosthesis *REEF™* (De Puy, Warsaw, IN, USA), allowing only for cementless stability (Fig. 3).

In some selected patients (polymicrobial infection or relevant comorbidities) receiving uncemented implants, an antibacterial coating was used. Surgeons chose either to cover the implant with the antibiotic-loaded hydrogel coating *DAC*® (Defensive Antibacterial Coating ®, Novagenit Srl, Mezzolombardo, Italy) just before the implantation (since it became available at our institution in 2014, or to use a silver-coated megaprosthesis (*PorAg*®, Link, Hamburg, Germany). Silver coatings were sometimes used also for the extramedullary portion of cemented stems and *DAC*® to protect the cementless cup used together with cemented stems.

When cemented implants were preferred, a vancomycin- and gentamicin-loaded cement (*Vancogenx*® cement; Tecres, Sommacampagna, Italy) was used.

Postoperatively, a standardized rehabilitation protocol was applied. Contact weight-bearing was allowed with limited active abduction for the first two weeks, to enable good soft tissue healing. Thereafter, increased progressive weight-bearing was carried out, avoiding also involuntary movements of the operated limb during sleep.

One-stage exchanges followed the same intraoperative steps, postoperative antibiotic therapy and rehabilitation of two-stage procedures.

### Microbiological methods

Removed implants and periprosthetic tissues were sent to the laboratory within two hours from sample collection. Before 2015, sonication was used to improve microbiological cultures [28]. Removed devices were completely covered by sterile saline in a container, then sealed and sonicated for 5 min (30 kHz, 300 W) at room temperature in an ultrasound bath (VWR, Milan, Italy).

Since 2015, before culture, all samples were treated with 0.1% w:v Dithiothreitol to free pathogen from biofilm [29, 30]. The eluate obtained after Dithiothreitol or sonication treatment was centrifuged and the pellet plated on chocolate agar, Mac Conkey agar, Mannitol Salt agar and Sabouraud agar and inoculated into Brain Heart infusion and Thyoglycollate broths. Plates were incubated for 48 h at 37 °C while broths were maintained at 37 °C for 15 days and daily checked for microbial growth. Aliquots from broths showing any turbidity were plated on Blood agar and, in case of Thyoglycollate, also on Schaedler agar. Microbial identification and antimicrobial susceptibility testing were carried out on Vitek2 system.

### Assessment

Each patient with a modular megaprosthesis was clinically and radiologically assessed (together with serial CRP measurement) in every scheduled follow-up at 1, 3, 6, 12, 24 months and then every 24 months according to our internal protocol. X-ray diagnoses of loosening were categorized upon Harris’ criteria [23]. Further assessments were done in case of minor o major implant failure or infection recurrence, when they became clinically evident. The average follow-up was 64 months (24–120), and no patient was lost.

We collected data on: megaprosthesis survival rate; infection eradication rate; revision rate; pain level measured with visual analogue scale (VAS) score; and functional status using MSTS at last follow up [31]. The MSTS provides a functional score on six items, scored from 0 to 5 points each (maximum score = 30): pain; walking ability; gait impairment; need of walking aids; overall function; and emotional acceptance [31]. Scores were considered excellent for MSTS > 20, satisfactory with MSTS between 10 and 20 and insufficient for MSTS < 10. A blinded surgeon collected the outcome data.

The primary endpoints of this study were: the infection healing rate, after a minimum 24-months follow-up, and the major revision rate. The infection was considered eradicated in case of negative clinical and laboratory parameters (CRP lower than MSIS definition thresholds), and according to Delphi criteria (no PJI-related death, healed wound with painless joint and without sinus tracts, no recurrence due to the same microorganism, no further reintervention for PJI recurrence) at 24 months [32]. Major revision was defined as a substitution of either the stem or the cup, while open reduction of dislocated MPs, either with or without liner exchange, were considered as minor revisions, because the latter are characterized by a shorter surgical time and reduced blood loss. The overall implant survival (survival free from infection recurrence and/or any surgical revision) was considered as secondary outcome.

### Statistical analysis

Statistical analysis was performed with SPSS Statistics 22.0 (IBM Corporation, Armonk, New York, US); variables were descripted through percentages, mean (range) or median (interquartile range, IQR). The implant survival was evaluated using Kaplan–Meier analysis [33], and Log rank to compare different groups (coated and uncoated MP, cemented and uncemented MP). Survival rate was expressed as percentage (95% confidence interval). Statistical significance was set at p < 0.05.

## Results

### Patients’ features, microbiology, surgical details

Clinical and radiological data were available for all patients. All cases had undergone from 2 to 6 previous interventions on the same joint (median 4, IQR 2–5). Femoral bone defects were classified as Paprosky type IIIB in 5 cases and Type IV in 16. The most frequent acetabular defect was Paprosky 2, and most patient received a standard or multihole revision cup. A single patient, showing a massive acetabular defect, was treated with a Burch-Schneider cage.

Patients’ demographics, preoperative data, microbiological results and follow up are available in Table 1.

**Table 1:** Patient demographics, diagnoses, cultures, follow up

Case	Sex	Age	Comorbidities	Diagnosis	Cultures	DTT?	Follow-up
1	F	55	Previous chondrosarcoma	PJI	Negative	–	120
2	M	63	–	PJI	*Pseudomonas aeruginosa*	Yes	120
3	F	57	–	PJI	Negative	–	120
4	F	85	HT, CAD	PJI	*Staphylococcus hominis*	No	72
5	F	76	MDD	PJI	Polymicrobial (*MRSA, MRSE*)	Yes	96
6	M	80	–	PJI	*MRSE*	Yes	96
7	F	56	Type 2 DM	PJI + Femoral septic NU	Polymicrobial (*Enterococcus faecalis, MSSA, Pseudomonas aeruginosa)*	Yes	96
8	M	60	–	PJI + Femoral septic NU	Polymicrobial (*Pseudomonas aeruginosa*, *Acinetobacter baumanii*, *MRSA*)	Yes	72
9	F	34	–	PJI (previously implanted MP)	*MRSE*	Yes	72
10	F	61	–	PJI + periprosthetic femoral fracture	*Morganella morganii*	Yes	72
11	M	67	–	PJI	*MRSA*	Yes	48
12	F	85	–	PJI	*MSSA*	No	48
13	F	83	MGUS, HT, Type 2 DM, thyroiditis	PJI	*MRSE*	Yes	48
14	M	54	HT, CAD, CKD, previous TB	PJI	Polymicrobial (*Staphylococcus lugdunensis, Enterococcus faecalis*)	No	48
15	M	53	Type 2 DM, HT	PJI	Polymicrobial (*MRSE, Propionibacterium acnes*)	Yes	48
16	F	72	HT, CKD	PJI	Polymicrobial (*Pseudomonas aeruginosa*, *MRSA*)	Yes	48
17	F	90	HT, CAD, DM, recurrent UTI	PJI	*Staphylococcus capitis*	Yes	24
18	F	81	Obesity, DM, recurrent UTI	PJI	*Klebsiella pneumoniae*	Yes	24
19	F	81	HT, CAD, CKD	PJI + Femoral septic NU	Polymicrobial (*Enterococcus faecalis, MRSE, Staphylococcus haemolyticus*)	Yes	24
20	F	63	Type 2 DM	PJI	*MSSA*	No	24
21	F	71	–	PJI	*Enterococcus faecalis*	No	24

*DTT* difficult-to-treat pathogens (according to Wimmer et al. [7] definition), *HT* hypertension, *CAD*  coronary artery disease, *MDD*  major depressive disorder, *DM*  Diabetes Mellitus, *MGUS* monoclonal gammopathy of unknown origin, *CKD* chronic kidney disease, *TB* tuberculosis, *UTI* urinary tract infections, *PJI* (chronic) periprosthetic joint infection, *NU* non-union, *MP* megaprosthesis, *MRSA* methicillin-resistant Staphylococcus aureus, *MSSA* methicillin-sensible Staphylococcus aureus, *MRSE* methicillin-resistant Staphylococcus epidermidis

The most frequently isolated pathogen was *Staphylococcus aureus* (7 patients), followed by *Staphylococcus epidermidis* (6 patients) and *Pseudomonas aeruginosa* (4 patients). A polymicrobial infection was found in 7 patients (33.3%) and negative intraoperative cultures in 2.

Twelve patients were affected by comorbidities. Seventeen were admitted for chronic PJI only. Patient no. 10 was admitted for a periprosthetic fracture around an infected THA. Patients no. 7, 8, 19 were admitted for septic non-unions of periprosthetic fractures with underlying PJIs.

Intraoperative details are resumed in Table 2.

**Table 2:** Surgical details

Case	One VS Two-Stage and Spacer type*	Interval between stages (days)	Implant	Coating
1	Two-stage (Vancogenx®)	84	Cemented Mega-C®	PorAg®
2	Two-stage (Spacer-G®)	161	REEF™	–
3	Two-stage (Spacer-G®)	98	REEF™	–
4	One-stage	–	REEF™	–
5	Two-stage (Spacer-G®)	81	REEF™	–
6	One-stage	–	REEF™	–
7	Two-stage (Vancogenx®)	196	REEF™	–
8	Two-stage (SO™S + Colistin)	184	Cemented Mega-C®	–
9	One-stage	–	Cementless Mega-C®	PorAg® + DAC®
10	Two-stage (Spacer-G®)	105	Cemented Mega-C®	DAC® (Acetabular Cup)
11	Two-stage (SO™S + G-Cl)	129	Cementless Mega-C®	PorAg®
12	Two-stage (Vancogenx®)	85	REEF™	–
13	Two-stage (Vancogenx®)	66	REEF™	–
14	Two-stage (SO™S + G-Cl)	126	Cementless Mega-C®	PorAg®
15	Two-stage (Vancogenx®)	91	Cementless Mega-C®	PorAg® + DAC®
16	Two-stage (SO™S + G-Cl)	94	Cementless Mega-C®	DAC®
17	Two-stage (Vancogenx®)	270	Cementless Mega-C®	PorAg®
18	Two-stage (Vancogenx®)	196	Cementless Mega-C®	PorAg® + DAC®
19	Two-stage (Vancogenx®)	93	Cemented Mega-C®	PorAg®
20	Two-stage (Vancogenx®)	120	Cementless Mega-C®	PorAg® + DAC®
21	Two-stage (Vancogenx®)	149	Cementless Mega-C®	DAC®

*SO™S + Colistin* colistin-loaded StageOne™ Select custom-made spacer, *SO™S + G-Cl* gentamicin- and clindamycin-loaded StageOne™ Select custom-made spacer, *MP* megaprosthesis, *LLD*  limb length discrepancy, *DAC®* Defensive Antibacterial Coating, *VAS*  Visual Analog Scale

All the REEF™ implants are cementless. *Spacer type, if used

In three cases only a one-stage exchange was performed. Out of 18 patients undergoing staged revisions, 5 needed an interim spacer exchange. We recorded 11 spacer dislocations (61%). The spacer instability required no further surgical treatment.

All patients finally underwent reimplantation. Seventeen patients received cementless megaprostheses, 12 of which were protected with an antibacterial coating (*PorAg*® and/or *DAC*®. Three out of the 4 cemented implants received a coating (*PorAg*® for the extramedullar femoral component or *DAC*® for the cementless acetabular cup). No intraoperative complication was reported.

### Infection recurrence

At a mean follow-up of 64 months (24–120), infection recurrence was observed in 2 patients (n°18 and n°21), who refused further surgeries and were treated with an antibiotic suppressive therapy, without further signs of implant loosening. Both had received a DAC®-coated MP 2 years before (Table 2). Nevertheless, both patients needed an open reduction of MP dislocation within 4 weeks after MPs reimplantation, and the resorbable DAC® coating was not applied again during this revision surgery.

**Fig. 4:**
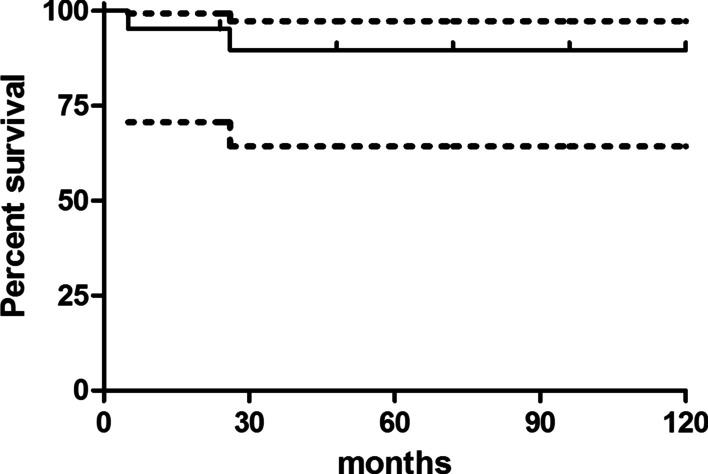
Kaplan Meier survival plot (outcome: infection-free survival). Only 10 patients reached a ≥ 5 year-follow up

The infection eradication rate was 90.5%, with a Kaplan Meier infection free survival of 95.2% at 2 years (95%CI 70.7–99.3) and 89.6% (95%CI 64.3–97.3) at 5 years. The Kaplan Meier curve takes into account that only 10 patients out of 21 had a ≥ 5 year-follow up (Fig. 4).

No statistically significant differences were found comparing the infection free survival and the major revision free survival and overall implant survivals either between the coated and uncoated groups, or between the cemented and cementless groups (Table 3).

**Table 3:** Log rank analysis

Outcome	Compared groups	Survival at 2 years (CI95%)	Survival at 5 years (CI95%)	p-value
Infection free survival	Coated	91.7% (53.9–98.8)	80.2% (40.2–94.8)	0.17
	Uncoated	100% (100–100)	100% (100–100)	
	Uncoated	100% (100–100)	100% (100–100)	0.22
	PorAg®	100% (100–100)	100% (100–100)	
	DAC®	100% (100–100)	66.7% (5.4–94.5)	
	PorAg® + DAC®	75% (12.8–96.1)	75% (12.8–96.1)	
	Cemented	100% (100–100)	100% (100–100)	0.50
	Cementless	94.1% (65–99.2)	87.4% (58.1–96.7)	
Major revision free survival	Coated	91.7% (53.9–98.8)	91.7% (53.9–98.8)	0.97
	Uncoated	100% (100–100)	88.9% (43.3–98.4)	
	Uncoated	100% (100–100)	88.9% (43.3–98.4)	0.29
	PorAg®	100% (100–100)	100% (100–100)	
	DAC®	66.7% (5.4–94.5)	66.7% (5.4–94.5)	
	PorAg® + DAC®	100% (100–100)	100% (100–100)	
	Cemented	75% (12.8–96.1)	75% (12.8–96.1)	0.19
	Cementless	100% (100–100)	92.3% (56.6–98.9)	
Overall revision free survival		83.35 (50.7–93.94)	83.35 (50.7–93.94)	

### Revision free survival, complications, functional outcome

**Fig. 5:**
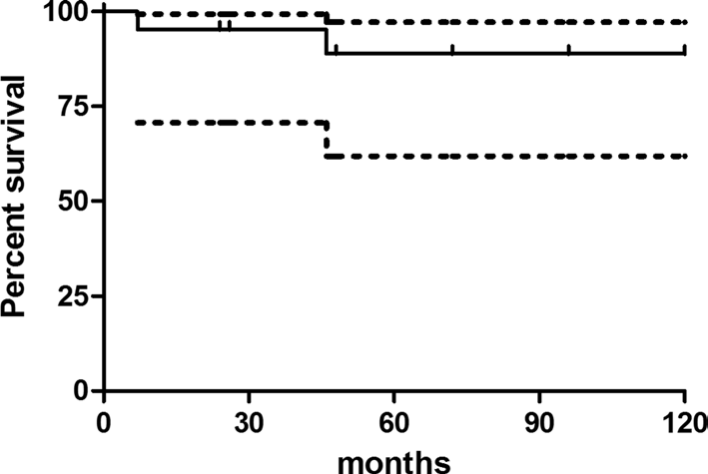
Kaplan Meier survival plot (outcome: major revision-free survival). Only 10 patients reached a ≥ 5 year-follow up

Two patients (9.5%) reported a mechanical implant failure, due to aseptic loosening of the cup (patient no. 4) or of the stem (patient no. 10), respectively 46 and 7 months after reimplantation. Both were treated with a revision of the failed implant component (major revision). The Kaplan–Meier survival of implants was 95.2% (95%CI 70.7–99.3) at 2 years and 89.6% (95%CI 64.3–97.3) at 5 years, considering major revisions as outcome measure (Fig. 5).

The most frequent complication was MP dislocation (8 patients, 38.1%), treated in 7 patients with open reduction with or without liner substitution using dual mobility components (minor revisions). One patient was treated with closed reduction only. No further dislocations were reported, but one patient has been using hip abduction orthosis since the reduction surgery. No other complication required additional surgery.

**Fig. 6:**
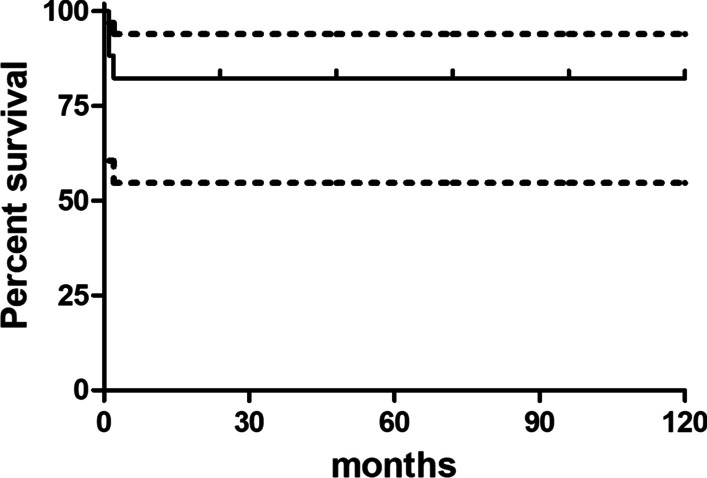
Kaplan Meier survival plot (outcome: overall implant survival). Only 10 patients reached a ≥ 5 year-follow up

The overall implant survival, considering both infection recurrences, major and minor revisions, was 83.35% (CI95% 50.7–93.94) at 2 and 5 years (Fig. 6) with an overall revision rate of 33.3%. In fact, 7 out of 21 patients underwent at least a revision; among them, two patients underwent both a minor and, later, a major revision, and further two patients underwent a minor revision and a septic failure due to infection recurrence, but they refused further revision surgeries.

The mean MSTS clinical score at last follow up was 20.6 (8–30), with excellent functional results in 12 patients and no pain in 18 patients (Table 4). More in detail: 9 patients permanently required two crutches for walking; 5 patients were able to walk with the support of a single cane, 7 patients did not require any walking support (1 of them used a permanent brace). Seven patients declared to be able to walk as preoperatively with no walking fatigue, 12 patients were able to walk outside for a variably reduced walking distance, 2 patients were very old and compromised by systemic diseases, so they were not able to walk outside, but could move at home without a wheelchair. Analyzing the gait quality, 2 patients had major cosmetic problems caused by severe limb length discrepancy, conditioning a minor functional deficit, 4 had no alterations, the others presented only minor cosmetic issues.

**Table 4:** Outcomes, complications, functional scores

Case	Infection recurrence	Complications and minor surgeries	Brace	Implant revisions	VAS (0–10)	MSTS Score (0–30)
1	No	–	No	–	0	27
2	No	LLD (− 2.5 cm)	No	–	1	23
3	No	–	No	–	0	27
4	No	Dislocation (open reduction and insert substitution)	No	1 (acetabular cup revision for aseptic loosening 2 years later)	0	17
5	No	Dislocation (open reduction and insert substitution), persistence of prior LLD (-2 cm)	No	–	0	16
6	No	–	No	–	0	25
7	No	–	No	–	0	21
8	No	–	No	–	0	19
9	No	–	No	–	0	30
10	No	Dislocation (open reduction and insert substitution)	No	2 (MP revision for aseptic stem loosening)	0	14
11	No	–	No	–	0	17
12	No	–	No	–	0	21
13	No	–	No	–	0	23
14	No	–	No	–	0	22
15	No	Dislocation (open reduction and insert substitution + larger size ceramic head)	No	–	0	28
16	No	Dislocation (closed reduction)	No	–	2	8
17	No	Wound dehiscence	No	–	0	17
18	Yes	Dislocation (open reduction and insert substitution)	No	–	5	8
19	No	Dislocation (open reduction and insert substitution)	Yes	–	0	19
20	No	–	No	–	0	29
21	Yes	Dislocation (open reduction)	No	–	0	21

*MP* megaprosthesis, *LLD*  limb length discrepancy, *VAS*  visual analog scale, *MSTS* Musculoskeletal Tumor Society

No patient showed signs of radiological loosening, dislocation or further osteolysis at last follow-up. Infection recurrences, revisions and functional outcomes are resumed in Table 4.

## Discussion

In our series of PJIs with severe femoral bone loss treated with MPs, we found an overall infection eradication rate of 90.5% at an approximately mean 5-year follow-up, the highest reported to date. This analysis confirms the efficacy of PFA in these cases. Nevertheless, it should be noticed that in our series only 10 patients had a ≥ 5 year-follow up, and about a quarter had a 2-year follow up only.

PJIs with severe bone defects are probably one of the worst scenarios faced by orthopedic surgeons today, and the standard of care is missing. Treatment is often demanding and, as in our case series, patients may undergo multiple complex operations, with high perioperative risks and significant costs for national healthcare systems [34]. Performing RTHA on these deficient bones, especially in the presence of ipsilateral knee prosthetic implants, constitutes a real challenge: standard revision implants are unsuitable in several cases [13, 35].

Recently, there has been a growing interest in the use of MP for non-tumoral indications, and heterogeneous series (periprosthetic fractures, massive bone defects, etc.) have been published [14, 35, 36]. Nevertheless, few previous studies showed the results of MPs for the treatment of PJIs [13, 14, 37–39]. Artiaco et al. published a series of 5 patients undergoing RTHA for PJIs with cemented modular MPs (4 two-stage cases and 1 one-stage); after a minimum 36-month follow-up, they observed infection recurrence only in the one-stage patient [37]. Ramappa et al. reported 6 cases of chronic knee PJIs treated with one-stage exchange of distal and/or total femur MP, at a minimum 18-month follow-up: 5 (80%) patients successfully completed the antibiotic treatment without infection recurrence, pain or reduced mobility [39]. Corona et al. published a series of 29 patients divided into 3 groups according to the type of MP they had received (proximal, distal o total femur arthroplasty); after a mean 48-month follow-up, they showed an infection healing rate of 82.8% (24 patients) [13]. Grammatopoulos et al. reported a series of 40 patients who underwent MP implantation in single (16 patients) or staged (24 patients) procedures. Nine patients received a silver-coated implant (Agluna®), six of which were implanted in one-stage surgeries [14]. Eradication of infection was achieved in 33 (83%). All seven patients with persistent or recurrent infection had been treated for polymicrobial PJI. Subsequent treatment included MP revision (n = 4) and debridement, antibiotics, and implant retention (DAIR) (n = 3). At a median follow-up of four years, four of these patients were on long-term antibiotic therapy. The authors did not specify whether patients with persistent infections had received a silver-coated implant or not. More recently, Döring et al. reported a mixed case series of MPs implanted for non-oncological purposes. Eleven PJIs were treated for PJI, and coating use was not mentioned: 3 were lost at follow up and 5 had a recurrence [38].

The choice between one- or two-stage procedures, in case of persistent PJIs after several failed surgeries, is a frequent matter of debate. In our experience, a two-step approach is likely preferable to reduce the risk of infection persistence, because staged procedures allow two clean-up interventions. However, the superiority of one technique over another is currently the subject of controversy [40, 41]. In this series, only three patients underwent one-stage exchange. For patient no. 4, one-stage surgery represented a protocol violation, as he was ≥ 80 years old. Nevertheless, as he was affected by coronary artery disease, he had a very high operatory risk, so we opted for a one stage surgery in order to spare him a double surgery within few months. This choice likely did not affect the overall implant survival, as the cup revision he underwent two years later was not due to infection recurrence, but to aseptic loosening.

In a study by Gomez et al., specifically designed to evaluate the clinical course of patients between the two stages of revision total hip or knee arthroplasties, the authors demonstrated that the 11.9% of patients needs an interim spacer exchange [42]. In our series, the 23.8% of patients (5 out of 18 receiving a staged surgery) underwent a spacer exchange for infection persistence before the definitive reimplantation. Our percentage was higher than the one reported by Gomez et al., probably because patients needing a MP (excluded from the cited study) usually have a massive infectious involvement of the femur. This could have led to a higher rate of infection persistence, and consequently to a higher rate of spacer exchange.

Persistence of infection after two-stage revision in the treatment of PJI remains a challenge. In our cohort, all patients finally underwent reimplantation, while Gomez et al. report that the 17.3% were never reimplanted [42]. It could be due to several factors, but a possible reason for this unsatisfactory outcome is the difficulty in evaluating persistent infection before second-stage surgery. A diagnostic gold standard test to determine the infection persistence at reimplantation in fact is still missing: to this end, the synovial leucocyte esterase strip test seems to be a promising intra-operative diagnostic tool, beyond serum CRP and ESR assays [43, 44].

We hypothesized that MPs should provide non-inferior infection eradication rates than standard-size implants in a PJI scenario. Beyond the possibility that larger metal surfaces have a higher colonization risk, the longer surgical time, the wider surgical approaches and the greater frequency of polymicrobial infections may rise the risk of infection persistence [19]. Despite all, our overall infection eradication rate (90.5%) was similar to other studies reporting RTHA with standard prostheses [13, 40]. It could be explained with the fact that PFA allows for a more radical surgery and bone debridement than RTHA, considering that 14 out of 21 patients had at least a difficult-to-treat microorganism isolated. Compared to standard revision surgery, PFA should be performed by surgeons experienced in bone infection management, after careful debridement and, when needed, extensive bone resections. Besides, these surgeries should be performed in referral centers only [13].

Furthermore, the possible protective role of coatings on infection recurrence should not be forgotten. The efficacy of silver-coated MPs in preventing infections has been highlighted in oncological patients, so much to become the gold standard for this patients’ subgroup [45]. Nevertheless, silver has the limitation to coat only the extramedullary portion of MPs. A fast-resorbable hydrogel coating, composed of covalently linked hyaluronan and poly-D,L-lactide (DAC®), was developed to avoid bacterial colonization in the first few hours after surgery, providing short-term local antibiotic delivery, while minimizing potential side effects and resistance development [46, 47]. This hydrogel can be used to coat even cups, ceramic heads and liners, so that we used it in combination with a silver-coated MP in 4 patients. A reason for the high infection eradication rate of our series could lie in the use of antibacterial coatings, which protect also patients with persistent positive cultures at the time of re-implantation. Nevertheless, the comparison between coated and uncoated implants survival, as well as between cemented and cementless MP, did not reach the level of statistical significance in this study. MPs used for non-oncological purposes are in fact rarely performed, and patients’ cohorts have often not enough power to perform reliable subgroup analyses. Furthermore, both patients who reported an infection recurrence after 2 years, had previously received a minor revision surgery within a month from the second stage surgery, because of MP dislocation. A possible MP contamination during this revision surgery cannot be excluded, considering that none of them received a novel application of the hydrogel coating, already resorbed at the time of revision surgery.

As previously reported, dislocations were the most frequent complication [15]. We reported 11 spacer dislocations. This high rate was expected, due to the extensive bone loss, the soft tissue disruption following multiple previous surgeries, and the lack of offset of the long-stem preformed spacers that were used in the study. Spacer dislocations were not treated, because of the temporary role of the spacer itself, and the intrinsic instability of these implants. Nevertheless, the use long-stem spacers gave the possibility to maintain a good femoral length and a better control of the operated limb, compared to the alternative options of not applying any spacer or using spacers with a standard short stem. Dislocations of definitive implants were less frequent, occurring in 8 cases. In the systematic review on proximal femoral arthroplasty in non-neoplastic conditions published by Mancino et al., the most common postoperative complication was dislocation, occurring in 74 out of 578 patients (12.8%), and requiring closed reduction in the 40.5% and reoperation in the 59.5% of cases [48]. Our dislocation rate was 23.8%, slightly higher than the value reported in the cited article. Several factors may have influenced our dislocation rate, most of all the soft tissues condition after previous surgeries (median: 4 surgeries) to treat infection persistence, the inability to achieve secure repair of the abductors to the MPs and inadequate soft tissue tension achieved postoperatively. Implant instability in fact depends on several factors, including: failure to restore the correct length, implant anteversion, type of liners employed, implant offset, cup positioning, head size, etc. Parvizi et al. suggested the use of constrained liners to prevent recurrent dislocations [1]. Other authors reported a dislocation rate of 37% even after special technical precautions, which included less than 30° cup inclination, reattachment of the abductor apparatus and limb-lengthening by about 1 cm [49]. Implant instability remains a main issue after PFA, and specific studies are needed to weight the relative role of all the variables, and to define a treatment algorithm. Given the intrinsic instability of these implants after several revision surgeries, we suggest using dual-mobility cups since second-stage surgery, and not only after a dislocation occurs, as suggested by the most recent literature [50].

Despite the dislocation rate, the overall satisfaction rate towards the treatment with MPs was good: the mean MSTS clinical score at last follow up was 20.6. These results are encouraging and strongly support this surgical option. This result is in line with the literature, reporting hip scores improvements with PFA [2, 5, 15]. Furthermore, we should not forget that these high-risk patients, as an alternative, could be treated only with resection arthroplasty or disarticulation. We suppose that the functional results guaranteed by PFAs are anyway far superior, compared to these non-reconstructive surgeries.

There is only one study on quality of life and patients’ satisfaction following MP implantation in PJI cases, but they do not report the use of antibacterial coatings and consider together knee, hip and total femur MPs [13].

### Limitations

This was a retrospective analysis, intrinsically providing a lower level of evidence than prospective studies. No control groups with PFA for different indications were available, because our unit is specialized in the treatment of orthopedic infections only. Finally, no power study was performed, and study sample size was limited to the rare patients receiving a surgical indication for PFA. This limited the reliability of log rank subgroup analyses. Furthermore, we found a highly variable interval between the two-stages, due to the organizational needs of our institution.

Unluckily, several heterogeneity factors may affect the results of clinical reports dealing with rarely indicated complex surgical reconstructions, including patients’ features and needs, isolated microorganisms and their spectrum of antimicrobial sensitivities, surgical techniques and biomaterials available, institutional organizational needs.

Nevertheless, there are very few works available in the literature, presumably due to the rarity of this revision, as well as to the relative novelty of this indication. Therefore, our results must be viewed with reserve, and larger comparative studies with longer follow-up are needed both to confirm long-term implant survival and how it could be influenced by coatings and cementation. Certainly, it should be considered that, in case of MP failure, some complex cases could end up in disarticulation, therefore patients should be adequately informed of all possible therapeutic strategies available.

## Conclusions

Our data suggest that PFA is useful for the treatment of PJI with severe bone loss, with results, in term of infection eradication, implant survival, functionality and patient satisfaction, in all similar to that of standard revision procedures.

## Data Availability

All data generated or analysed during this study are included in this published article (tables).

## Abbreviations

PJI/PJIs: Periprosthetic joint infection/infections
RTHA: Revision total hip arthroplasty
MP/MPs: Megaprosthesis/megaprostheses
PFA: Proximal femoral arthroplasty
*DAC*®: Defensive antibacterial coating
CRP: C reactive protein
VAS: Visual Analog Scale
THA: Total hip arthroplasty
DAIR: Debridement, antibiotics, implant retention
